# Gender differences in specialty preference and mismatch with real needs in Japanese medical students

**DOI:** 10.1186/1472-6920-10-15

**Published:** 2010-02-11

**Authors:** Yoshiharu Fukuda, Tadanari Harada

**Affiliations:** 1 Department of Community Health and Medicine, Yamaguchi University School of Medicine, Yamaguchi, Japan

## Abstract

**Background:**

The shortage of doctors and maldistribution among specialties are of great concern in the Japanese health care system. This study investigated specialty preference in medical students of one university, and examined gender differences and compared their preference with real needs.

**Methods:**

We conducted a self-administered questionnaire including specialty preference in all students of one medical university. Preference was assessed by the five-level probability of their future choice: 1 = very low, 2 = low, 3 = moderate, 4 = high, and 5 = very high. The proportion of 4 or 5 was calculated as the preference rate. The real needs (magnitude of doctor shortage) in the prefecture were drawn from two different surveys. The relationship between the sex-specific preference rate by specialty and real needs was assessed by Spearman's correlation coefficient.

**Results:**

Internal medicine showed the highest preference rate, followed by general surgery, pediatrics, and emergency medicine. There was no significant correlation between the preference rates of men and women (r = 0.27, p = 0.34). The preference rates for general surgery, orthopedics, neurosurgery, and emergency medicine were significantly higher in men than in women, while those of obstetrics & gynecology, pediatrics, and dermatology were significantly higher in women. The magnitude of doctor shortage by specialty from two surveys were significantly correlated with the total preference rate and men's preference rate (r = 0.54 to 0.74), but not with women's preference rate (r = 0.06 and 0.32).

**Conclusions:**

This study elucidated not only gender differences in specialty preference but also the relationship to real needs. Critical gender differences and mismatch with real needs were found in women. In addition to traditional gender roles and insufficient support for women's participation in Japan, gender differences and mismatch influence the current and future maldistribution of specialties. Systematic changes in the working environment in medical society are required to solve these problems.

## Background

The so-called "medical crisis" is recently of great concern in Japanese society. The shortage of doctors has led to closure of medical facilities and restriction of access to sufficient medical services [1,2]. It has been pointed out that the governmental policy to prevent an increasing number of doctors in order to control medical expenditure has resulted in a doctor shortage and a medical crisis [1,2].

In addition to the absolute shortage of doctors, maldistribution of doctors by specialties and geographical areas are urgent problems in the Japanese health care system. A governmental report stated that obstetrics & gynecology, pediatrics, and anesthesiology have suffered a more severe shortage than other specialties [3]. Among the closures of departments of hospitals in recent years, obstetrics & gynecology and pediatrics have shown the largest numbers [4]. Other specialties such as general surgery also show a decreased number of doctors [2].

In order to correct the maldistribution of doctors by specialty, factors that influence the choice and preference of specialty of doctors and medical students should be identified. Previous studies in other countries identified several factors related to the choice and preference, including sex, career opportunities, prestige, income and others [5-12]. Among them, most of the studies found gender differences in doctors' and medical students' choice and preference [5,6,8,11,12]. Also in Japan, the gender ratio varies according to the specialty [13].

Another focus of this study was whether the choice and preference match the real needs for doctors. The relationship between the magnitude of shortage in specialties and preference of medical students will influence the future allocation of specialties. However, little is known about the relationship between real needs and the preference by specialty, since real needs (magnitude of doctor shortage) by specialty are difficult to elucidate.

We have estimated the magnitude of doctor shortage by specialty at the local level through two different surveys [14]. These surveys quantified the number of doctors needed by specialty in one prefecture (Yamaguchi prefecture) [14]. On the other hand, we conducted a survey in medical students at a university located in this prefecture [14]. Linking the data of real needs and students' specialty preference makes it possible to examine the relationship between real needs and students' preferences. This study investigated specialty preference in medical students of one university, and examined the gender differences and compared their preferences with the real needs.

## Methods

### Medical student survey

We conducted a survey in medical students of Yamaguchi University School of Medicine. The self-administered questionnaire was disseminated and collected in the classrooms for 1st to 4th grade students and via the administration office for 5th and 6th grade students, who participate in bedside learning at hospitals. The survey was conducted from October to December, 2008. This survey aimed to elucidate not only specialty preference but also reasons of selection of internship hospitals, opinions on community health, and others. The questionnaire with questions used in this paper is attached as Additional file 1.

The questionnaire included a question on specialty preference. In terms of the specialties listed in Table 1, preference was assessed on the basis of five levels of probability of future choice: 1 = very low, 2 = low, 3 = moderate, 4 = high, and 5 = very high. The proportion of 4 or 5 was calculated as the preference rate.

**Table 1:** Basic characteristics of responders: comparison between men and women

Variables			Men		Women		Total		p-values^a^
Responders (response rate)		N (%)	303	(84.1%)	190	(93.6%)	493	(86.9%)	0.35
Age (years)		Mean ± S.D.	23.3 ± 3.6		22.7 ± 3.2		23.1 ± 3.4		0.08
Year	1st	N (%)	53	(17.5%)	25	(13.2%)	78	(15.8%)	0.16
	2nd	N (%)	47	(15.5%)	43	(22.6%)	90	(18.3%)	
	3rd	N (%)	45	(14.9%)	37	(19.5%)	82	(16.6%)	
	4th	N (%)	60	(19.8%)	36	(18.9%)	96	(19.5%)	
	5th	N (%)	57	(18.8%)	28	(14.7%)	85	(17.2%)	
	6th	N (%)	41	(13.5%)	21	(11.1%)	62	(12.6%)	
Transfer students^b^		N (%)	20	(6.6%)	12	(6.3%)	32	(6.5%)	0.91
Students from Yamaguchi prefecture^c^		N (%)	93	(30.7%)	52	(27.4%)	145	(29.4%)	0.42

^a ^Comparison between men and women with chi-square test or t-test

^b ^Transfer students who had passed the entrance examination for bachelor degree students

^c ^Students from Yamaguchi prefecture

### Surveys of doctor shortage

We used data on doctor shortage from two different surveys.

One survey was conducted by the Yamaguchi Medical Association in 2007 [15]. The survey mainly examined the working conditions of employed doctors. The participation rate was estimated to be about 55%. Using the macrofile of this survey, we estimated the number of doctors required to eliminate overwork (more than 48 hours per week) [14], for each specialty. The results were extrapolated from the results of the Physician, Dentist and Pharmacists Survey in 2006 [13], which is routinely conducted by the national government, to calculate the number of doctors in hospitals in Yamaguchi prefecture.

The other survey was originally conducted by directors of departments in all hospitals in Yamaguchi prefecture [14]. We asked them to supply the present number of doctors and the required number of doctors by specialty in each hospital, and accumulated the numbers into total numbers in the prefecture.

The detailed results of these surveys are presented in our previous report [14].

### Analyses

The association between preference rates of men and women, and between preference rates and the magnitude of doctor shortage from the two surveys were examined by Spearman's correlation coefficients. The significance level was 0.05.

## Results

Among 567 medical students, 493 students responded and completed the questionnaire (response rate = 86.9%). The rate of women (190/203 = 93.6%) was higher than that of men (303/360 = 84.1%), but the difference was not statistically significant. The rates ranged from 64.6% in the 6th grade to 97.8% in the 2nd grade.

Table 1 shows the basic characteristics of the responders who were analyzed, with comparison between men and women. There was no difference in age, year (grade), proportion of transfer students (who had passed the entrance examination for bachelor degree students), and proportion of students who came from Yamaguchi prefecture.

Table 2 summarizes the specialty preferences of medical students, and the numbers of required doctors and the shortage of doctors in Yamaguchi prefecture from the two surveys.

**Table 2:** Specialty preference of medical students and number of required doctors in one prefecture from two surveys

	Preference rate (%)^a^			p-value^b^	Employed doctor survey		Hospital director survey	
				
Specialty	Men	Women	Total		Required	Lacking	Required	Lacking
Internal medicine	50.7	56.1	52.8	0.24	854.2	128.2	797.5	211.3
General surgery	31.3	16.0	25.4	<0.01	335.5	60.5	287.6	45.3
Pediatrics	17.3	31.7	22.7	<0.01	92.5	11.5	89.4	28.6
Psychiatry	12.6	18.0	14.7	0.10	161.0	11.0	150.3	34.4
Orthopedics	23.0	9.0	17.6	<0.01	184.8	30.8	167.3	55.9
Neurosurgery	14.7	8.5	12.3	0.04	98.5	18.5	89.3	26.2
Obstetrics & gynecology	4.0	26.3	12.7	<0.01	93.1	18.1	92.9	29.7
Ophthalmology	9.4	16.4	12.1	0.20	56.3	6.3	47.6	11.9
Otolaryngology	9.0	13.2	10.6	0.14	60.8	10.8	44.8	11.8
Dermatology	8.6	19.0	12.7	<0.01	34.1	4.1	32.5	13.6
Urology	2.7	7.9	4.7	<0.01	85.8	16.8	74.1	16.4
Radiology	9.0	9.0	9.0	0.99	84.7	8.7	74.0	17.2
Plastic-cosmetic surgery	5.3	13.2	8.4	<0.01	8.7	0.7	15.1	3.2
Anesthesiology	12.0	15.9	13.5	0.22	80.5	14.5	75.3	29.0
Emergency medicine	22.3	14.4	19.3	0.03	33.0	9.0	37.0	31.4

^a ^Proportion of students who responded "4: high probability" or "5: very high probability" of future choice (from scores 1 to 5)

^b ^Comparison between men and women with chi-squared test

Internal medicine showed the highest preference rate for both men and women (total 52.8%). For men, general surgery (31.3%), orthopedics (23.0%), emergency medicine (22.3%), and pediatrics (17.3%) followed. For women, pediatrics (31.7%), obstetrics & gynecology (26.3%), dermatology (19.0%), and psychiatry (18.0%) followed.

Concerning the shortage of doctors, internal medicine showed the largest shortage in both surveys. Although there were some differences in the magnitude of the shortage by specialty between the two surveys, the magnitude of the shortage by specialty showed a strong correlation (r = 0.75, p = 0.001).

**Figure 1:**
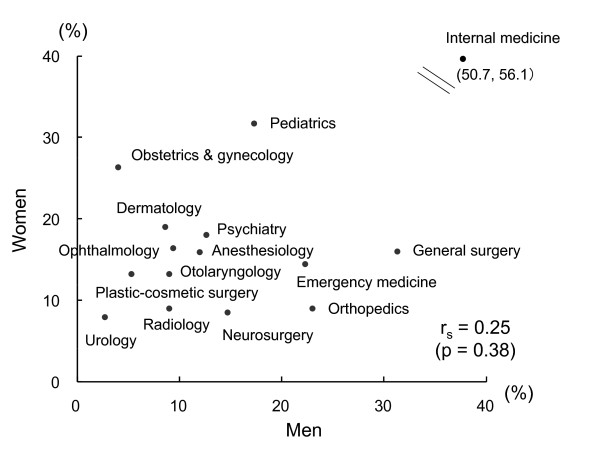
**Relationship of sex-specific specialty preferences of medical students in one university in Japan**. r is Spearman's correlation coefficient.

Figure 1 shows a scatter diagram of sex-specific preference rates. There was no significant correlation between men's and women's preference rates (r = 0.25, p = 0.38). General surgery, orthopedics, emergency medicine, and neurosurgery showed much higher preference rates for men than for women, while obstetrics & gynecology and pediatrics showed much higher preference rates for women than for men [14].

**Figure 2:**
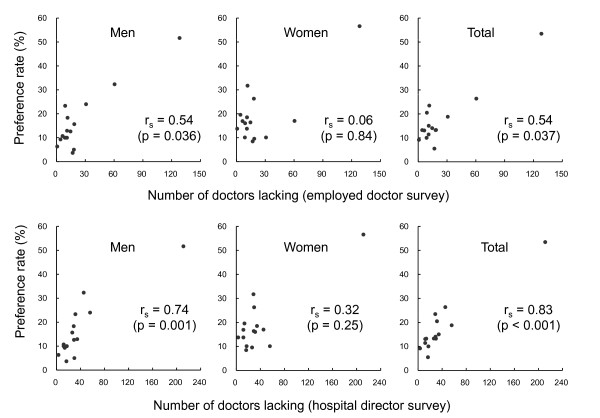
**Relationship between shortage of doctors and specialty preferences of medical students in one prefecture in Japan**. r is Spearman's correlation coefficient.

Figure 2 shows scatter diagrams of preference rates and the magnitude of doctor shortage by specialty. In both surveys, men showed a significant relationship (r = 0.54 and 0.74), while women did not (r = 0.06 and 0.32). For total students, the specialty rate was significantly correlated with the magnitude of the shortage of doctors (r = 0.54 and 0.83).

## Discussion

This study demonstrated gender differences in specialty preference among medical students, and the relationship between specialty preference and real needs (the magnitude of doctor shortage) by specialty. As a result, this study showed significant gender differences and mismatch between specialty preference and real needs in female students.

Gender differences in specialty choice and preference in medical students and doctors are common across countries [5,6,8,11,12]. Previous studies in other countries demonstrated that the specialties generally preferred by women are pediatrics and obstetrics & gynecology [5,6,10,11]. Our study showed that Japan has a similar pattern of women's preferences. Some factors such as control of lifestyle and work-life balance were identified as being related to women's specialty preference and choice [8-12].

We examined whether the specialty preference in medical students matched the real specialty needs, and demonstrated a relationship between specialty preference and real specialty needs. As a result, although the overall preference was correlated with the real needs, women's preference was not. Considering the recent situation of health care in Japan, we focused on the following two groups of specialties facing problems.

The first group includes obstetrics & gynecology and pediatrics. These specialties are preferred and chosen dominantly by women, and the level of preference is consistent with the magnitude of doctor shortage. However, obstetrics & gynecology and pediatrics are faced with heavy workloads and a doctor shortage [16-18]. It is possible that the heavy workloads in these specialties will increase in accordance with the increasing number of female doctors because of their limited working hours, due to their physical characteristics and life course including pregnancies and child care.

The second group includes general surgery, orthopedics, and emergency medicine. These specialties are preferred and chosen dominantly by men, and the level of preference is lower than the magnitude of doctor shortage. Heavy workloads and uncontrollable lifestyle partly explain the lower preference for these specialties in female doctors [8,19]. Previous studies in other countries showed that prestige and career opportunity were factors promoting surgical specialties, while lifestyle and perceived quality of the patient/physician relationship were preventive factors [20,21]. The absolute shortage of doctors in these specialties will be increased by the increasing proportion of female doctors. Unless the working environment in health care systematically changes, the shortage of doctors in these specialties is not expected to be solved.

A few limitations of this study should be mentioned. First, the real need, that is, the magnitude of doctor shortage by specialty, is generally difficult to estimate. We used the data of two surveys. Since these surveys estimated the number of required doctors based on different methodologies [14], the number of required doctors showed some differences between the two surveys. However, the correlation of the magnitude of the shortage between the two surveys was significantly high (r = 0.75). Therefore, we concluded that the relative levels of doctor shortage among specialties seem to have been reliably identified. Second, this study examined the situation in only one prefecture and in one university in the prefecture, and it would be important to ascertain whether the results of this study are consistent with those of other situations and the whole country. Since the number of doctors per population in Yamaguchi prefecture is similar to the nationwide level [13], it seems that the results of this study did not show a particular case. Lastly, specialty preference might be influenced by various factors such as the student's grade and demographic characteristics. In terms of the effects of education at the university and the relationship with such as their age, hometown, experiences before entrance of the university (including characteristics of the high school), or parental occupation, more detailed analysis is required.

To solve the problem of shortage and maldistribution among specialties, it is necessary to discuss policy implications. However, this problem appears to be very difficult to solve and there is no one solution. Briefly, there are two possible solutions: change of preference and change of needs.

Concerning changing the preference, excessive gender differences might be undesirable. Previous studies in other countries have shown that gender differences are associated with factors such as heavy workload and work-family balance including childcare [9]. The factors associated with the gender differences in specialty preference in Japan should be elucidated in more detail. Improving the working environment with consideration of these factors would contribute to changing medical students' and doctors' preferences.

As shown in previous studies, prestige and income are important factors influencing specialty preference [10,20,22]. The insurance program in Japan does not sufficiently consider the prestige of specialties, including doctors' fees [2]. The prestige and incentives should be increased to lead medical students and young doctors into specialties requiring long-term training and a heavy workload.

We should also consider the changing needs. It is not expected that the number of doctors, especially in male-dominant specialties such as general surgery and emergency medicine, will remarkably increase. Strategies not only to increase the number of doctors, but also to establish suitable working environments under a decreasing number of doctors should be discussed. The key is to improve the working conditions for female doctors, to increase the number of female doctors in male-dominant specialties (e.g., surgery) and to decrease the workload in female-dominant specialties (e.g., obstetrics & gynecology and pediatrics). Moreover, generalists including family doctors and highly skilled paramedical professionals will be expected to have roles supporting the shortage in certain specialties.

Lastly but importantly, supportive environments for female doctors should be encouraged. It has been pointed out that the participation of women in society in Japan is far from satisfactory [23]. This problem in Japanese society also applies to the health care setting. Indeed, a report on female Japanese doctors demonstrated that they suffered from poor work-life balance and that their retirement and layoff resulted from difficulties with childbirth and child-rearing [24]. Systematic changes in working environments including gender roles are required to solve the problems of shortage, overwork, and maldistribution of doctors in Japan.

## Conclusions

We elucidated gender differences in specialty preference of medical students at one university and the relationship between preference and real needs (shortage of doctors) in one prefecture in Japan. Critical gender differences and mismatch with real needs were found in women. In addition to traditional gender roles and insufficient support for women's participation in Japan, gender differences and mismatch influence the current and future maldistribution of specialties. Systematic changes in the working environment in medical society are required to solve these problems.

## Competing interests

The authors declare that they have no competing interests.

## Authors' contributions

YF is the primary author who was responsible for conceiving the research idea, designing the study, collection of data, analysis and interpretation of the results and writing of the draft and final manuscript. He is also the corresponding author. TH participated in designing the study, collection of data, and interpretation of the results. YF and TH read and approved the final manuscript.

## Pre-publication history

The pre-publication history for this paper can be accessed here:

http://www.biomedcentral.com/1472-6920/10/15/prepub

## Supplementary Material

Additional file 1 Questionnaire on specialty preference Click here for file
