# Supplementary figures and images for: Gender differences in specialty preference and mismatch with real needs in Japanese medical students
Source: BMC Med Educ. 2010 Feb 11;10:15. doi: 10.1186/1472-6920-10-15 (PMC2834693; doi:10.1186/1472-6920-10-15)

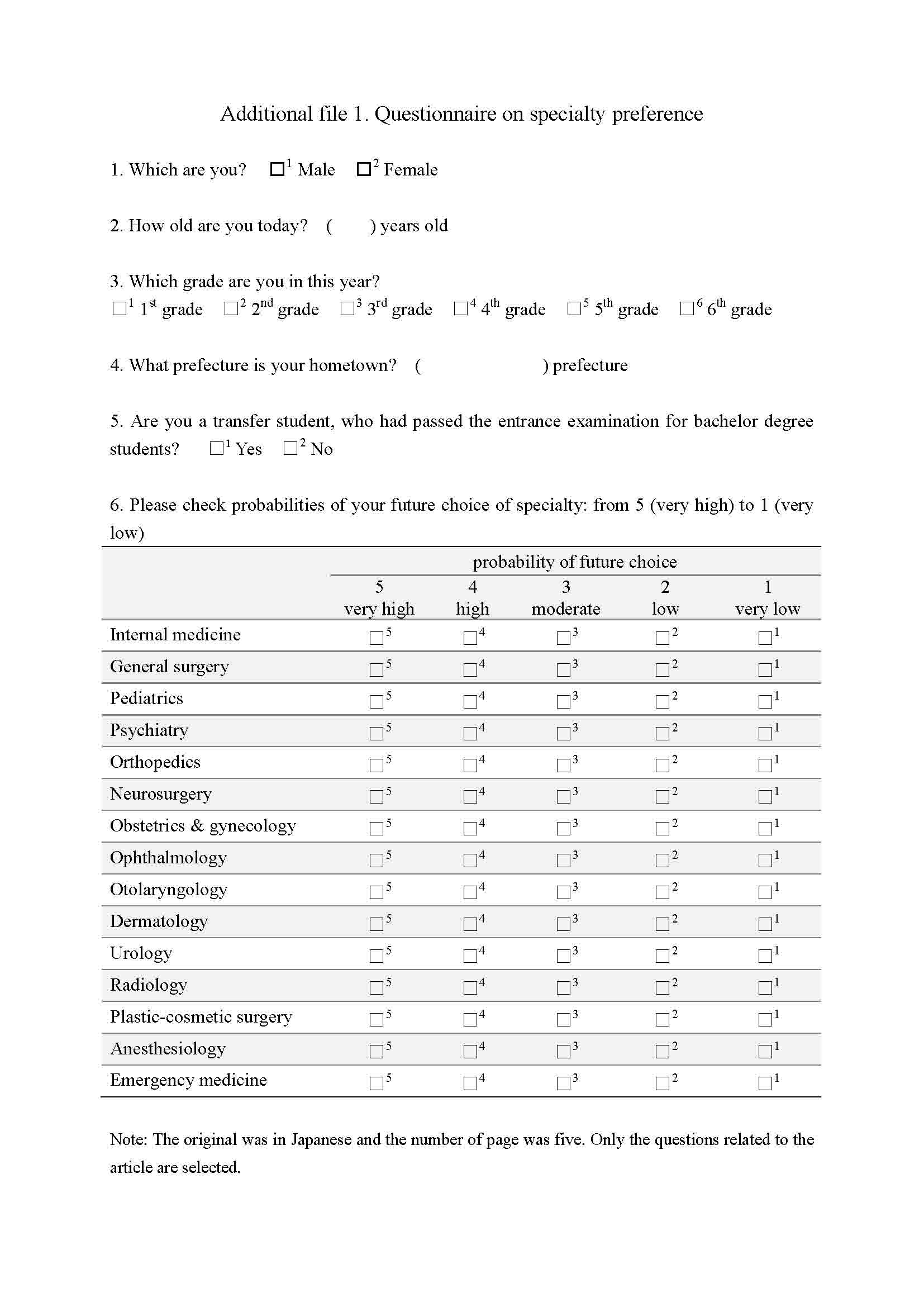

Supplement: Additional file 1 — Questionnaire on specialty preference [file 1472-6920-10-15-S1.JPEG]
